# Longitudinal study of single-pulse TMS in infants with perinatal brain injury: safety and feasibility

**DOI:** 10.3389/fnhum.2025.1686054

**Published:** 2025-10-15

**Authors:** Kellie M. Collins, Cameron P. Casey, Ellen N. Sutter, Paige DeGrave, Danielle Gauthier, Arun Karumattu Manattu, Hung-Shao Cheng, Ryan M. McAdams, Raghavendra Rao, Michael K. Georgieff, Bernadette T. Gillick

**Affiliations:** ^1^Division of Developmental Pediatrics & Rehabilitation Medicine, Department of Pediatrics, Waisman Center, University of Wisconsin-Madison, Madison, WI, United States; ^2^Pediatric Neuromodulation Laboratory, Waisman Center, University of Wisconsin-Madison, Madison, WI, United States; ^3^Department of Family Medicine and Community Health, University of Minnesota, Minneapolis, MN, United States; ^4^Division of Neonatology & Newborn Nursery, Division of Global Pediatrics, Department of Pediatrics, University of Wisconsin-Madison, Madison, WI, United States; ^5^Division of Neonatology, Department of Pediatrics, Masonic Institute for the Developing Brain, University of Minnesota, Minneapolis, MN, United States; ^6^Division of Neonatology, Department of Pediatrics, Department of Obstetrics, Gynecology and Women's Health, Center for Neurobehavioral Development, Masonic Institute for the Developing Brain, Institute of Child Development, University of Minnesota, Minneapolis, MN, United States

**Keywords:** cerebral palsy, perinatal brain injury, transcranial magenetic stimulation (TMS), motor-evoked potential (MEP), noninvasive brain stimulation (NIBS), pediatric neuromodulation, safety, feasibility

## Abstract

**Introduction:**

Perinatal brain injury is a leading cause of cerebral palsy. Single-pulse transcranial magnetic stimulation (spTMS) provides a non-invasive method for investigating motor pathway development; however, data on the safety and feasibility of its repeated use in infants are limited. This study provides the first longitudinal evaluation of the safety, tolerability, and feasibility of spTMS in infants with perinatal brain injury.

**Methods:**

Twenty infants with perinatal brain injury (corrected age 3–25 months) participated in 46 spTMS sessions while awake. Safety and tolerability were systematically assessed using heart rate, respiratory rate, and the Modified Behavioral Pain Scale (MBPS). Feasibility was quantified by session completion, participant retention, and acquisition of motor-evoked potentials (MEPs) from bilateral wrist flexors.

**Results:**

Across 2,527 pulses, no adverse events occurred. Physiological measures and MBPS scores remained stable from pre- to post-stimulation. Analyzable electromyography (EMG) was obtained in 100% of sessions, with MEPs successfully elicited in 44/46 sessions (95.7%) across 19/20 infants (95%). A high longitudinal retention rate (85%) further demonstrated excellent protocol acceptability.

**Discussion:**

These findings establish a safe, reproducible framework for longitudinal spTMS in a vulnerable infant population. This methodological advance enables future investigations into neuroplasticity and corticospinal tract development after early brain injury, with the potential to yield biomarkers that guide the timing and targets of early interventions.

## Introduction

Cerebral Palsy (CP), a motor disorder commonly caused by perinatal brain injury, is one of the leading causes of motor disability in childhood, significantly impacting movement, balance, and coordination (Berger et al., 2002; Rosenbaum et al., 2007). Despite recent advances in the early diagnosis of CP (Novak et al., 2017), critical questions remain about how early brain injury alters the neurodevelopmental processes that shape motor outcomes. One key area of interest is the development of the corticospinal tract (CST), the primary motor pathway responsible for voluntary movement, which undergoes dynamic refinement during the first 2 years of life (Evarts, 1968; Lemon, 2008). Perinatal brain injury can disrupt this trajectory, contributing to an increased risk of CP (Eyre et al., 2007; Holmefur et al., 2010; Cahill-Rowley and Rose, 2014; Kowalski et al., 2019), with variability in motor outcomes and early intervention needs (Gangwani et al., 2023). While changes in corticospinal development are believed to contribute to these outcomes, the ability to measure the functional integrity of these pathways reliably in infancy remains limited.

In typical infancy, bilateral CST projections innervate both hemispheres, followed by activity-dependent pruning that leads to predominant contralateral control (Eyre et al., 2001; Martin, 2005; Eyre et al., 2007; Gordon et al., 2013). Perinatal brain injury can disrupt this refinement process, producing atypical CST configurations such as retained ipsilateral or absent contralateral projections—patterns associated with worse motor outcomes (Eyre et al., 2007; Kuhnke et al., 2008; Holmström et al., 2010; Staudt, 2010; Gordon et al., 2013; Friel et al., 2014; Smorenburg et al., 2017; Rich et al., 2020; Gutterman and Gordon, 2023). While some studies suggest that preserved contralateral organization supports more favorable recovery, the functional implications of retained ipsilateral projections are less clear (Kuhnke et al., 2008; Friel et al., 2021; Gangwani et al., 2023). Understanding how CST development is altered following early brain injury is especially important during infancy, when heightened neuroplasticity may offer a time-limited window for targeted intervention (Lemon, 2008; Friel et al., 2014; Wachs et al., 2014).

Single-pulse transcranial magnetic stimulation (spTMS) provides a noninvasive method for probing CST integrity and cortical excitability *in vivo* (Kowalski et al., 2019). When magnetic pulses are applied to the primary motor cortex (M1), they elicit motor-evoked potentials (MEPs) in peripheral muscles, offering direct insight into the functional responsiveness of descending motor pathways (Hallett, 2007; Sutter et al., 2024). While spTMS has been widely used in older children, its implementation in infants remains limited due to concerns about safety, tolerability, and feasibility (Chen et al., 2017; Narayana et al., 2017; Kowalski et al., 2019; Nemanich et al., 2019; Saiote et al., 2022; Sutter et al., 2024). Establishing safe, age-appropriate protocols for spTMS is critical to advancing early neurodevelopmental research and translating recent discoveries in CST maturation into clinical applications.

Emerging evidence suggests that spTMS is well tolerated in infants and holds promise for studying motor system disruption following early brain injury (Tekgul et al., 2020; Antczak, 2021; Sutter et al., 2024). Pioneering work by Eyre and colleagues demonstrated the feasibility of using TMS to investigate early CST maturation during infancy (Eyre et al., 2001; Eyre et al., 2007). A recent review by Sutter et al. (2024) identified 23 studies that used spTMS in infants, although only 11 reported outcomes related to safety or tolerability. Critically, these studies broadly interpret the absence of reported adverse events as evidence of safety—an inference that lacks the rigor of systematic evaluation using structured, quantitative assessments. Most studies relied on observational measures and provided minimal documentation of standardized safety protocols, which limits generalizability and regulatory guidance. The International Federation of Clinical Neurophysiology offers general TMS safety recommendations (Rossi et al., 2021), but these do not specifically address infant populations.

The present study is built upon more than a decade of pediatric noninvasive brain stimulation (NIBS) research, drawing on foundational safety guidelines from the broader field (Frye et al., 2008; Rossi et al., 2009; Krishnan et al., 2015; Allen et al., 2017; Zewdie et al., 2020; Rossi et al., 2021; Metelski et al., 2024) and a dedicated line of research from our group (Gillick et al., 2015; Chen et al., 2017; Gillick et al., 2018; Keller-Ross et al., 2019; Kowalski et al., 2019; Nemanich et al., 2019; Kowalski et al., 2020; Saiote et al., 2022; Sutter et al., 2024). Our initial work in infants, Nemanich et al., provided the first structured report on spTMS safety and feasibility in a cohort with perinatal stroke (Nemanich et al., 2019). That foundational study created an infant-specific framework using systematic observational outcomes, such as infant state and discrete stress codes, alongside basic physiological monitoring. Subsequent studies from our lab further confirmed the stability of the cardiovascular response to spTMS (Keller-Ross et al., 2019) and documented safe application even in infants with complex medical conditions, such as implanted cardiac devices (Kowalski et al., 2020). This body of work collectively demonstrated that spTMS was well-tolerated and feasible, but underscored that safety had primarily been assessed through observational reports and single-metric physiologic data. While large-scale prospective studies on NIBS safety in children are emerging, dedicated protocols and quantitative data for preverbal infants remain scarce (Zewdie et al., 2020).

While our foundational research and a recent review (Sutter et al., 2024) confirmed the promise of spTMS, a critical gap was recognized: the lack of comprehensive, quantitative safety data collected across multiple developmental stages. To move from establishing feasibility to creating a scalable, reproducible methodology, a more rigorous approach was needed. This study was therefore designed to directly address the limitations of prior work by (1) implementing a larger, longitudinal design to track outcomes over the first 2 years of life; (2) incorporating the first quantitative analysis of multiple physiological measures (heart rate, respiratory rate) using robust linear mixed-effects models; and (3) introducing a validated behavioral distress scale (Modified Behavioral Pain Scale [MBPS]) to systematically and objectively assess infant tolerability. Regarding the specific methods used to assess feasibility, while this study uses MEP detection as a key feasibility metric, its presence is not interpreted as direct evidence of CST integrity. Instead, MEP acquisition is treated as a methodological benchmark to support future investigations into motor system development and neuroplasticity in this important population.

Therefore, the primary aim of this study was to establish the safety, tolerability, and overall feasibility of longitudinal spTMS in infants with perinatal brain injury. We hypothesized that the procedures would be safe and well-tolerated, as demonstrated by stable physiological measures and behavioral distress scores. As a quantitative measure of feasibility, our secondary aim was to assess methodological success through session completion rates and the acquisition of analyzable electromyography (EMG) data. Finally, as an exploratory objective, we investigated potential relationships between MEP detection and clinical or procedural factors, such as infant age, injury laterality, and stimulation intensity.

## Methods

### Setting and participants

spTMS sessions were conducted at the Waisman Center, University of Wisconsin-Madison. Eligible infants were those with a corrected age at study entry between term and 24 months with a radiologically confirmed unilateral or bilateral perinatal brain injury. Corrected age was calculated for pre-term infants (born < 37 weeks’ gestation) by subtracting the number of weeks they were born before 40 weeks from their chronological age; infants born at or after 37 weeks retained their chronological age. The resulting interval was then expressed in whole months and remaining weeks for reporting. Brain injuries included perinatal stroke, neonatal hemorrhagic or thrombotic stroke involving the motor cortex and/or subcortical structures, intracranial hemorrhage involving the motor cortex and/or subcortical white matter, periventricular leukomalacia (PVL), or hypoxic–ischemic encephalopathy (HIE). Injury laterality was determined from radiological reports. An injury was classified as unilateral (left or right) only if the report indicated no damage to the contralateral hemisphere. Cases with any reported injury to both hemispheres, regardless of extent or asymmetry, were classified as bilateral. Infants were excluded if they had unrelated neurological or metabolic disorders, disorders of cellular migration and proliferation, acquired traumatic brain injuries, or contraindications to MRI or spTMS. Complete inclusion and exclusion criteria are detailed in Saiote et al. (2022).

### Study design

This prospective, longitudinal observational study examined the safety, tolerability, and feasibility of performing spTMS with infants aged 0–25 months (corrected for gestational age) with radiologically confirmed perinatal brain injuries. Each participant completed between one and four spTMS sessions, depending on enrollment timing. Each session was scheduled at one of four predefined visits: V1 (3–6 months), V2 (12 months ±1 month), V3 (18 months ±1 month), and V4 (24 months ±1 month). Each session included assessments of both hemispheres while acquiring EMG responses bilaterally within the same session.

This work is part of a larger, multimodal investigation integrating magnetic resonance imaging (MRI) and motor behavioral assessments to examine neurodevelopment in this infant cohort (Saiote et al., 2022). The overarching study is funded by the National Institutes of Health/National Institute of Child Health and Human Development and the National Institute of Neurological Disorders and Stroke (7R01HD098202–02). It is registered on ClinicalTrials.gov (NCT05013736) and approved by the Institutional Review Board at the University of Wisconsin-Madison. Informed written consent was obtained from legal guardians prior to participation.

### spTMS setup and procedure

spTMS (Magstim 200^2^, Magstim, United Kingdom) was used to noninvasively stimulate the primary motor cortex (M1) in each hemisphere separately. Anatomical targeting of the M1 region was guided by individual T1-weighted MRI scans, which were reconstructed into three-dimensional brain models using a frameless stereotactic neuro-navigation system (Brainsight, RRID: SCR_009539, Rogue Research, Montreal, Quebec, Canada). For the seven sessions in which a study-specific scan was unsuccessful because the infant was unable to fall or remain asleep for the duration of the non-sedated MRI, age-matched templates were used for localization (Fonov et al., 2009); prior clinical MRIs were not used for targeting. A lightweight reflective head tracker was gently secured to the infant’s forehead using skin-safe, disposable adhesive pads. The tracker was then registered using anatomical landmarks (nasion, tip of the nose, bilateral preauricular notches). The hand-knob region of M1 was visually identified on the 3D brain model and marked within the Brainsight environment as the stimulation target with a specified 45-degree coil angle off the nasal-inion line.

**Figure 1:**
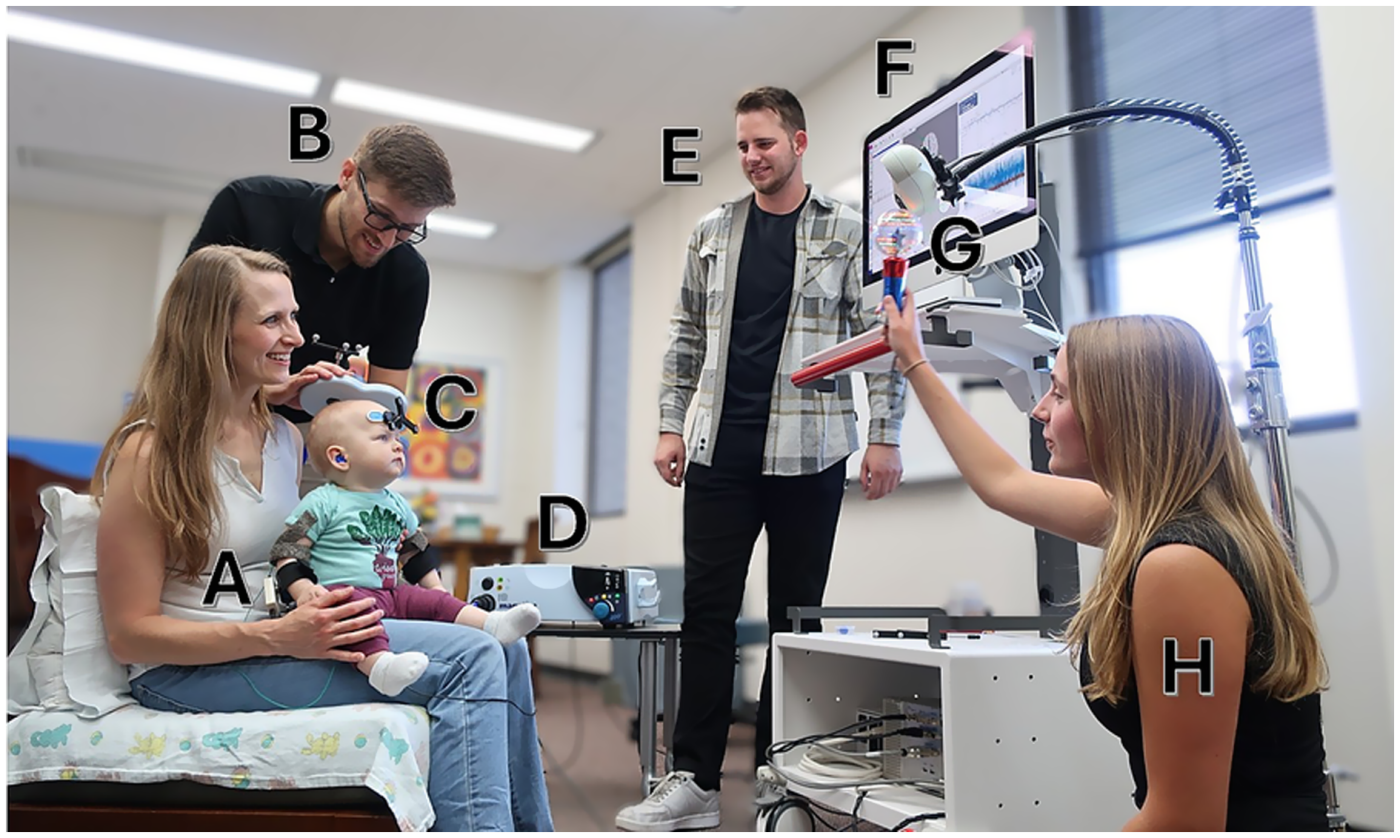
General setup of a single-pulse transcranial magnetic stimulation (spTMS) session. A pilot study participant **(A)** is seated with a caregiver while one team member **(B)** delivers spTMS using a Magstim D70^2^ coil **(C)** connected to a Magstim 200^2^ stimulator **(D)**. Another team member **(E)** operates the Brainsight neuronavigation software, which provides real-time coil tracking **(F)** via an infrared camera **(G)**. A third team member **(H)** engages the infant with age-appropriate activities. An additional safety monitor is present but not pictured. Written informed consent for the publication of this photograph was obtained from the adult individual(s) and the parent or legal guardian of the child. The photograph depicts a pilot session and does not show an individual enrolled in the present study.

A standardized, team-based setup was employed for all assessments to ensure safety, maintain engagement, and support procedural accuracy. The team included four members: a TMS operator responsible for coil positioning and stimulation, a neuro-navigation operator, a behavioral safety monitor attending to the infant’s well-being, and a team member dedicated to infant engagement and distraction. Caregivers were directly involved in supporting infant positioning and comfort throughout the session. This coordinated setup is illustrated in Figure 1.

Positioning was tailored to the infant’s developmental stage and the caregiver’s preference. Infants aged 3–5 months or those with limited head or trunk control were supported in seated, supine, standing, or cradled positions with caregiver assistance. Older infants sat or stood with or without caregiver support, including sitting on a caregiver’s lap. To facilitate stillness and optimize engagement, sessions incorporated caregiver interaction, toys, visual stimuli, and team-based support strategies.

Surface EMG responses were recorded bilaterally from wrist flexor muscles using the Brainsight integrated EMG system. Two gel-based electrodes, trimmed to ~3 cm diameter, were placed on the anteromedial forearm, with dampened ground straps secured to the upper arms. Established infant protocols were followed during electrode application to ensure signal consistency (Chen et al., 2017). Self-adhering wraps and skin-safe markers were used to ensure consistent placement and minimize displacement. Time-locked EMG signals were recorded using a 2-channel EMG device with a 16–470 Hz bandwidth (Bai et al., 2023). Data was obtained at a sampling rate of 3 kHz, capturing activity from 50 ms prior to 150 ms following each TMS pulse. All signals were high-pass filtered and stored digitally for offline analysis.

Stimulation was delivered using a figure-of-eight Magstim D70^2^ coil manually positioned over the targeted M1 site. Pulse intensity began at 50% maximum stimulator output (MSO) and was increased in 5% increments. An intensity was considered to have successfully elicited a response if it produced at least three MEPs within five trials. If this criterion was not met, the intensity was increased until either the criterion was met or 80% MSO was reached. If no MEPs were elicited at the initial ‘hand-knob’ target, the operator delivered pulses to the four surrounding quadrants within a 1 cm radius to search for a more optimal site. This exploratory step was particularly important in cases where an age-matched template was used for neuronavigation or when the hand-knob was not clearly visible due to the nature of the brain injury. A maximum of 100 pulses was delivered per hemisphere to balance a thorough assessment with infant comfort. Additional details on the stimulation protocol are provided in Saiote et al. (2022).

Following the assessment of the first hemisphere, the procedure was repeated for the contralateral hemisphere. All sessions were performed while the infant was awake, with breaks provided as needed to accommodate feeding and sleep routines.

### Safety monitoring and procedures

All safety monitoring and procedures were conducted in accordance with a comprehensive protocol previously established and published by our group to ensure the safe administration of spTMS in pediatric populations (Gillick et al., 2018). Each single pulse was administered with a minimum 10-s interstimulus interval between pulses to minimize the risk of adverse events (Saiote et al., 2022). A multi-step safety approach was implemented that included caregiver screening, physiological monitoring, auditory protection, behavioral pain assessment, and structured team oversight.

**Figure 2:**
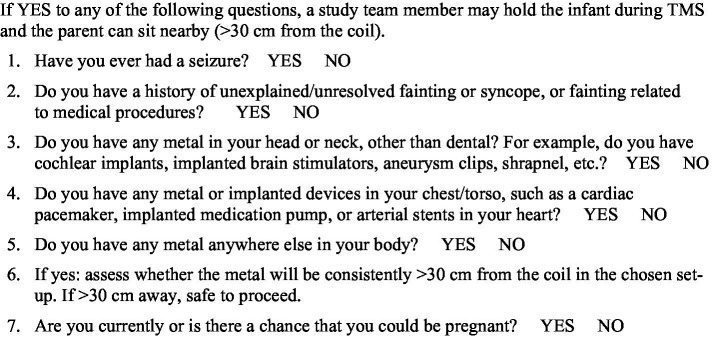
Parent/guardian screening for transcranial magnetic stimulation.

Before each session, caregivers completed a structured screening protocol to ensure it was safe for them to hold their child while spTMS was administered (Figure 2). Throughout the session, trained personnel recorded physiological parameters immediately before and after stimulation. Heart rate (HR) was measured by palpating the brachial artery, and respiratory rate (RR) was assessed by observing chest movements. Both were recorded over 15 s and extrapolated to beats or breaths per minute. Pain and discomfort were assessed using the Modified Behavioral Pain Scale (MBPS), a validated observational tool for procedural distress in infants. The MPBS rates facial expression, crying, and body movement on a scale from 0 (no distress) to 10 (severe distress; Crellin et al., 2018a). To standardize the assessment, ratings were taken at two specific time points: immediately before the first TMS pulse and immediately after the final pulse. All ratings were performed by a team member independent of the TMS operator to reduce bias. To ensure consistency across the multiple personnel responsible for scoring, all raters were trained using an apprenticeship model. This training required each new rater to first observe a full spTMS session scored by an experienced team member. Following this observation, a debriefing was held to review the application of the MBPS criteria and to confirm the new rater’s understanding before they performed assessments independently. As an additional training resource, a library of previously recorded spTMS sessions was available for optional review, allowing new raters to compare their practice scores with those recorded during the actual sessions. Although timing and scoring were standardized and raters completed an apprenticeship training process, a formal inter-rater reliability statistic for MBPS was not computed in this cohort.

To mitigate auditory exposure from the TMS coil discharge, both infants and nearby adults wore hearing protection. Infants were fitted with silicone ear putty, and caregivers and team members used standard foam earplugs. A brief behavioral hearing screen was conducted before and after each session by shaking a rattle out of the infant’s line of sight on either side and monitoring for a startle or orienting response.

If the infant showed signs of distress, stimulation was paused and the infant’s ability to continue with the protocol was reassessed by the team. Caregivers were contacted within 24 h of the session for follow-up. A study physician reviewed all outcomes, and any adverse events would have been reported per protocol. No adverse events occurred in this study.

### Data analysis

All statistical analyses were conducted in R version 4.5.0 (2025-04-11 ucrt) using RStudio.

### Physiological and behavioral safety analysis

Descriptive statistics, including means, standard deviations, medians, and interquartile ranges (IQRs), were calculated for heart rate (HR), respiratory rate (RR), and Modified Behavioral Pain Scale (MBPS) scores.

To test the null hypothesis that spTMS had no effect on physiological or behavioral outcomes, we used linear mixed-effects models (LMEMs) to account for the nested data structure (repeated sessions within infants). Using the *lme4* package in R, we fitted separate models for HR, RR, and MBPS scores (Bates et al., 2015). The models were specified as: *Outcome ~ Time + (1 | Subject) + (1 | Subject: Visit)*, where *Time* was a fixed effect representing pre- vs. post-stimulation measurement. Model assumptions were evaluated using the *DHARMa* package in R (Hartig, 2024) through visual inspection of scaled residual plots and quantitative tests for uniformity and dispersion. Effect sizes for these pre-post comparisons were calculated using Cohen’s d.

To test the null hypothesis that stimulation intensity and duration had no effect on physiological or behavioral outcomes, we again used LMEMs. We modeled post-session HR, RR, and MBPS scores as a function of two separate predictors: the total number of pulses delivered and the maximum stimulation intensity (%MSO). To ensure model convergence, these models required a simplified random-effects structure with only a random intercept for the participant, specified as: *Outcome ~ Predictor + (1 | Subject)*. As before, model assumptions were evaluated using the *DHARMa* package by inspecting scaled residual plots and performing quantitative tests for uniformity and dispersion. F-tests, *p*-values, and partial eta-squared (η_p_^2^) effect sizes were obtained using a Type III ANOVA with a Kenward-Roger approximation.

As a secondary sensitivity analysis, and to account for the smaller sample sizes within age-based subgroups where parametric assumptions may not be met, we also examined within-session changes using non-parametric tests. We used paired Wilcoxon signed-rank tests to assess pre- to post-session changes in HR, RR, and MBPS for each visit (V1-V4). The null hypothesis for these tests was that the median difference between pre- and post-session scores was zero. A rank-biserial correlation (r) was calculated as the measure of effect size. For descriptive purposes, we also computed the mean and standard deviation of change scores (post-pre) within each age stratum.

### MEP scoring and consensus determination

Three raters (KMC, CPC, HSC) independently reviewed EMG trials and scored each for the presence of a MEP using a 5-point ordinal confidence scale: −2 (high confidence MEP absent), −1 (moderate confidence MEP absent), 0 (ambiguous or artifact-obscured), +1 (moderate confidence MEP present), and +2 (high confidence MEP present). We defined the MEP latency window as 10–50 ms to be consistent with prior pediatric studies (Koh and Eyre, 1988; Eyre et al., 2001; Kowalski et al., 2019) and to account for potential variability stemming from factors such as individual differences, developmental stage, and brain injury (Koh and Eyre, 1988; Cantone et al., 2019; Cleland et al., 2021). A score of +2 was assigned only if all of the following were met: (1) the putative MEP onset was between 10 and 50 ms of the TMS pulse (Kowalski et al., 2019); (2) its peak-to-peak amplitude exceeded 50 μV (Lieberman et al., 2006); and (3) the putative MEP signal was visually distinct from baseline EMG activity (Chowdhury et al., 2024). A score of −2 was assigned when none of these criteria were satisfied. Intermediate scores (−1, 0, +1) reflected partial or uncertain fulfillment of criteria.

Scoring was performed independently, with trial order randomized for each rater. Raters were blinded to the hemisphere of stimulation, muscle laterality, and all participant-level data. Consensus was defined as agreement between at least two raters on the same score, with the third rater differing by no more than one point. The modal value was recorded as the consensus score. For each hemisphere-hand combination, MEP classification was based on the distribution of consensus scores across all EMG trials within that session. Combinations were classified as MEP-positive (POS) if at least one trial received a consensus score of +1 or +2. If no trials were rated +1 or +2, but at least one trial received a score of 0, the combination was documented as negative with uncertainty (NEG-U). If all trials received consensus scores of −1 or −2, the combination was classified as MEP-negative with high confidence (NEG-D, *D = definite*).

For datasets with low interrater reliability, defined as an intraclass correlation coefficient across raters less than 0.6, a structured consensus review was conducted. All three raters met (in person or virtually) to re-examine trials without consensus. Prior to reviewing, original scores were archived. A final consensus score was assigned only when all three raters agreed. Trials that had previously met consensus were not re-evaluated. This process enhanced consistency and minimized individual rater bias in classifying infant MEP responses. The reliability of this specific scoring and consensus method has been formally validated, demonstrating good inter-rater reliability for infant MEPs (ICC = 0.784; Casey et al., 2025).

### MEP data quantification and feasibility

The primary feasibility outcome was the successful acquisition of MEPs. To assess this, we quantified the total number of EMG trials and the proportion of these trials scored as positive based on consensus criteria. A chi-squared test was used to compare the proportion of positive trials obtained from contralateral versus ipsilateral pathways. These trial-level outcomes were then used to assign one of three session-level classifications to each hemisphere-hand combination: MEP-positive (POS), MEP-negative with uncertainty (NEG-U), or MEP-negative with high confidence (NEG-D), as detailed in the *MEP Scoring and Consensus Determination* section.

We categorized the session-level outcomes (POS, NEG-U, NEG-D) in two ways. Our descriptive analyses retained all three categories to provide a nuanced view of MEP detection rates across clinical subgroups. In contrast, our inferential logistic mixed-effects models required greater statistical power per group, so the NEG-U and NEG-D categories were collapsed into a single MEP-negative (NEG) category. This dichotomization (POS vs. NEG) was chosen to sensitively test for the presence of any MEP, aligning with the study’s primary feasibility aim and ensuring all data contributed to the final models. Modeling at this level of the hemisphere-hand combination, rather than the individual trial, allowed for a direct test of factors influencing CST responsiveness while appropriately accounting for non-independence of repeated pulses within a session.

### Statistical modeling of MEP outcomes

To examine factors associated with MEP acquisition, we fitted a series of logistic mixed-effects models using the *glmer* function from the *lme4* package (44). All models included *Participant* as a random intercept to account for repeated measures from the same infant. Statistical significance for each fixed effect was determined using a likelihood-ratio test, which compared the full model containing the predictor of interest to a null model lacking that effect.

This modeling approach was used to test associations between MEP presence and several primary factors of interest: age *(MEP_presence ~ Visit_Age)* and injury laterality (i.e., left, right, or bilateral; *MEP_presence ~ Injury_Laterality*). Additionally, we modeled the binary MEP outcome as a function of the maximum stimulation intensity reached during the corresponding session *(MEP_presence ~ Max_MSO)*. As a secondary outcome, we applied the same method to examine the absence of contralateral MEPs from the injured hemisphere(s). While the presence of MEPs does not directly index CST integrity, prior work suggests that the absence of contralateral projections may reflect atypical corticospinal development and is often associated with poorer motor outcomes in older children (Eyre et al., 2007; Holmström et al., 2010; Staudt, 2010; Gordon et al., 2013; Friel et al., 2014; Smorenburg et al., 2017) Specifically, we tested for the association between contralateral MEP absence and injury laterality *(Contralateral_MEP_absent ~ Injury_Laterality)* and directly compared MEP detection rates from injured versus uninjured hemispheres *(MEP_presence ~ Hemisphere_Status)*. This latter analysis was restricted to contralateral pathways only. Given that bilateral corticospinal projections are typical in early infancy, we did not attempt to classify or interpret ipsilateral responses.

## Results

### Participant characteristics

Twenty participants (5 females, 15 males) were included in the analysis. Corrected ages at the time of spTMS ranged from 3 months 1 week to 24 months 4 weeks (mean ± standard deviation (SD) = 11 months 3 weeks ± 7 months 1 week). By visit, corrected mean ages were: 4 months 2 weeks ± 1 month 0 weeks at V1, 11 months 3 weeks ± 0 months 3 weeks at V2, 18 months 1 week ± 0 months 3 weeks at V3, and 24 months 1 week ± 0 months 4 weeks at V4. Each infant completed between one and four spTMS sessions: 8 infants completed one session, 2 completed two sessions, 6 completed three sessions, and 4 completed all four sessions (Table 1).

**Table 1:** Participant demographics and clinical characteristics.

Characteristic	Value
Age at spTMS Assessment (months)	
Mean (SD) Overall, *n* = 20	11 months 3 weeks (7 months 1 week)
Range Overall, *n* = 20	3 months 1 week - 24 months 4 weeks
Mean (SD) at V1 (3–6 Month), *n* = 19	4 months 2 weeks (1 month)
Mean (SD) at V2 (12-Month), *n* = 12	11 months 3 weeks (4 weeks)
Mean (SD) at V3 (18-Month), *n* = 9	18 months 1 week (3 weeks)
Mean (SD) at V4 (24-Month), *n* = 6	24 months 1 week (4 weeks)
Sex, n (%)	
Male	15 (75%)
Female	5 (25%)
Race, n (%)	
American Indian or Alaska Native	0 (0%)
Asian	0 (0%)
Black or African American	2 (10%)
Native Hawaiian or Other Pacific Islander	0 (0%)
White	18 (90%)
Unknown	0 (0%)
Does not specify	0 (0%)
Ethnicity, n (%)	
Hispanic or Latino	0 (0%)
Not Hispanic or Latino	20 (100%)
Unknown	0 (0%)
Does not specify	0 (0%)
Injury Laterality, n (%)	
Left Hemisphere	3 (15%)
Right Hemisphere	4 (20%)
Bilateral	13 (65%)
Injury Type, n (%)*	
Ischemic Infarct (INF)	7 (35%)
Hemorrhage (HEM)	13 (65%)
Periventricular Leukomalacia (PVL)	3 (15%)
Hypoxic–Ischemic Encephalopathy (HIE)	4 (20%)
Cerebral Palsy Diagnosis, n (%)**	8 (40%)
Topography (*n* = 6)***	
Hemiplegia	5 (83%)
Quadriplegia	1 (17%)
Motor Type (*n* = 7)***	
Spastic	5 (71%)
Dyskinetic	1 (14%)
Hypotonic	1 (14%)
Rural–Urban Commuting Area (RUCA) Codes, n (%)	
Metropolitan Areas	14 (70%)
Micropolitan Areas	3 (15%)
Rural Areas	3 (15%)
Area Deprivation Index (ADI) National Percentile	
Median (IQR)	40.5 (24)
Area Deprivation Index (ADI) State Decile	
Median (IQR)	3 (3.5)

Data are presented as mean (standard deviation; SD), median [interquartile range; IQR], or number (%). Age at spTMS assessment is expressed in months and weeks. Injury laterality was classified as unilateral (left [L] or right [R]) or bilateral (B). Injury types include ischemic infarct (INF), hemorrhage (HEM), periventricular leukomalacia (PVL), or hypoxic–ischemic encephalopathy (HIE); participants could be counted in multiple categories. Socioeconomic factors include Rural–Urban Commuting Area (RUCA) codes (metropolitan, micropolitan, rural) and the Area Deprivation Index (ADI), shown as a national percentile and state decile. *Diagnoses are not mutually exclusive; totals exceed the number of participants due to overlapping injury types. **At the time of the most recent study visit. ***Topography and motor type classifications were not available for all participants with CP. Percentages are based on available data (*n* = 6 for topography, *n* = 7 for motor type).

Patterns of brain injury and CP diagnosis varied across participants. The brain injury was identified as unilateral in 35% (7/20) of participants, with damage confined to either the left (15% [3/20]) or right (20% [4/20]). The remaining 65% (13/20) of infants had bilateral injuries. Brain injuries were categorized as ischemic infarct (INF; 35%, 7/20), hemorrhagic (HEM; 65% [13/20]), PVL (15% [3/20]), and HIE (20% [4/20]), with 35% of infants (7/20) classified under multiple categories. At the most recent study visit, 8 participants (40%) had received a formal CP diagnosis; 12 (60%) had not received a diagnosis or were awaiting further evaluation. Among those with CP, topography data were available for 6 infants: hemiplegia was most common (*n* = 5), followed by quadriplegia (*n* = 1). Motor type classifications were available for 7 participants: spastic CP (*n* = 5) was most common, followed by dyskinetic (*n* = 1) and hypotonic (*n* = 1).

Demographic characteristics are summarized in Table 1. The majority of participants were White (90% [18/20]) and non-Hispanic (100% [20/20]). Most resided in metropolitan areas (70% [14/20]), with the remainder from micropolitan (15% [3/20]) and rural (15% [3/20]) regions, based on Rural–Urban Commuting Area (RUCA) codes (47). Socioeconomic context, assessed using the Area Deprivation Index (ADI), showed a median state decile of 3 (IQR = 3.5; range: 1–9) and a median national percentile of 40.5 (IQR = 24; range: 3–86), indicating a demographically diverse sample.

### Session distribution and participation

Based on their timeline in participation, infants completed between one to four spTMS sessions, with a mean inter-session interval of 228 days (~7.5 months), median = 217 days, range = 150–421 days. In total, 46 sessions were conducted with 20 participants: 19 at V1, 12 at V2, 9 at V3, and 6 at V4. Fewer sessions occurred at later timepoints due to staggered enrollment; younger participants had not yet reached the older milestones.

In addition, attrition also occurred due to common factors in longitudinal studies, including loss to follow-up, caregiver preference, medical status changes, or illness. One family elected to withdraw after their child’s physician confirmed he was exceeding all developmental milestones. The family expressed confidence in his progress and wished to provide the study opportunity to another family with potentially greater needs. Despite the aforementioned factors, 85% (17/20) of eligible participants returned for at least one follow-up visit, indicating strong longitudinal retention and supporting the feasibility of repeated neurophysiological assessment in this population.

### Safety and tolerability outcomes: behavioral and physiological responses

**Figure 3:**
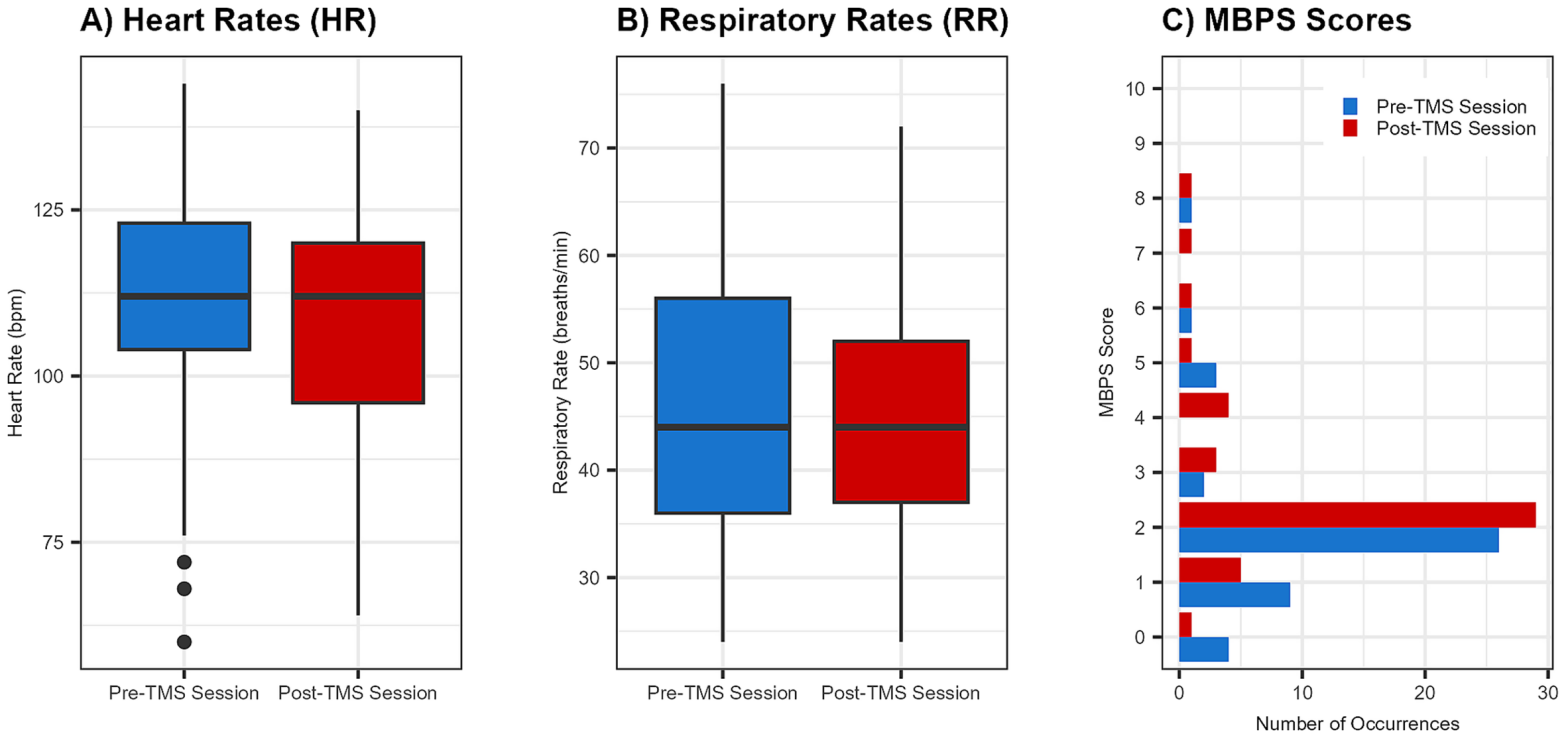
Group changes in physiological and behavioral metrics pre- and post-spTMS sessions. Boxplots illustrate pre- and post-session values for **(A)** heart rate (HR) in beats per minute (bpm) and **(B)** respiratory rate (RR) in breaths per minute. A bar graph **(C)** displays the frequency of each Modified Behavioral Pain Scale (MBPS) score s (0 = no distress, 10 = severe distress). For all plots, pre-session data are shown in blue and post-session data are in red. For boxplots, the horizontal line indicates the median.

**Figure 4:**
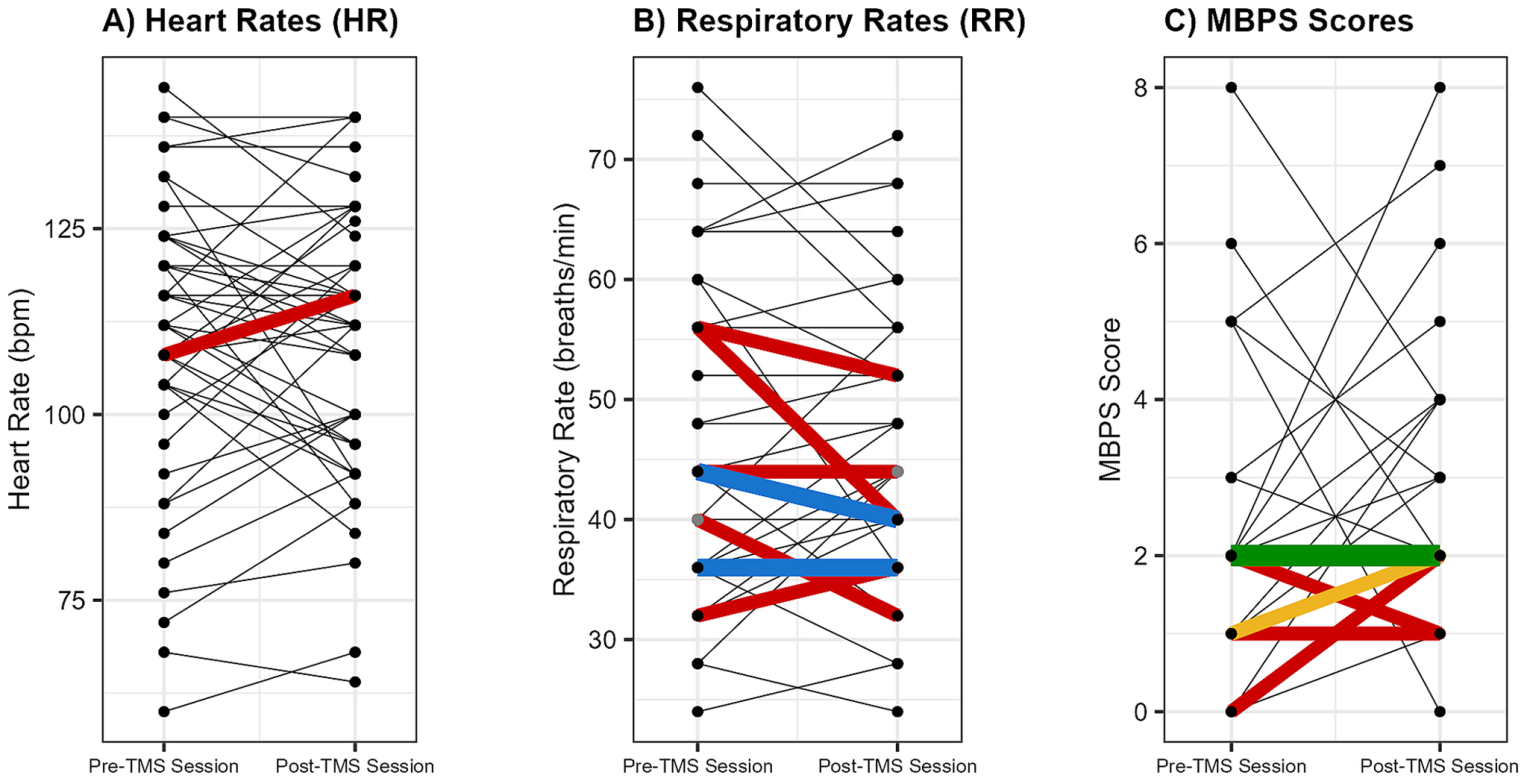
Individual changes in physiological and behavioral metrics pre- and post-spTMS sessions. Individual pre- and post-session changes are shown for **(A)** heart rate (HR; beats per minute [bpm]), **(B)** respiratory rate (RR; breaths per minute), and **(C)** Modified Behavioral Pain Scale (MBPS) scores (0 = no distress, 10 = severe distress). Each line connects the pre- and post-values from a single session. The line color indicates the frequency of that exact pre-to-post change across all sessions: black (occurred once), red (twice), blue (3 times), yellow (5 times), and green (20 times).

Across the 46 sessions, a total of 2,527 single TMS pulses were delivered, with no adverse events reported. Pre- and post-session hearing screens were completed for all infants, with no observed changes in auditory responsiveness. Stimulation was administered with a median interstimulus interval of 25.45 s. Overall, physiological (HR, RR) and behavioral (MBPS) responses remained stable from pre- to post-session (Figures 3, 4). Descriptive and inferential statistics by visit are reported in Table 2.

**Table 2:** Descriptive and inferential statistics for physiological and behavioral measures across age groups.

Measure	Visit number	Sessions, n	Pre mean (SD)	Post mean (SD)	Mean difference (SD)	*p*-value	Effect size (*r*)
HR (bpm)	V1	19	118.9 (18.9)	116.3 (19.2)	−2.6 (11.8)	0.32	0.26
	V2	12	101.3 (17.8)	106.3 (17.0)	5.0 (10.7)	0.18	0.41
	V3	9	107.1 (14.0)	106.2 (15.0)	−0.9 (18.8)	1.00	0.02
	V4	6	102.0 (23.6)	98.7 (17.1)	−3.3 (23.2)	0.92	0.09
RR (breaths/minute)	V1	19	55.4 (12.0)	52.2 (11.4)	−3.2 (10.7)	0.26	0.25
	V2	12	40.7 (5.9)	43.0 (4.9)	2.3 (4.3)	0.11	0.49
	V3	9	37.8 (8.5)	37.8 (10.0)	0.00 (8.0)	0.78	0.10
	V4	6	36.7 (7.8)	37.3 (6.5)	0.7 (6.9)	0.85	0.27
MBPS	V1	19	2.0 (1.4)	2.5 (1.31)	0.5 (1.6)	0.13	0.41
	V2	12	1.7 (0.7)	2.4 (1.9)	0.8 (1.8)	0.17	0.41
	V3	9	2.6 (2.2)	2.9 (1.7)	0.3 (2.2)	0.68	0.17
	V4	6	2.5 (2.1)	1.8 (1.0)	−0.7 (2.5)	0.71	0.13

Descriptive statistics (mean, standard deviation) are presented for pre- and post-session heart rate (HR; beats per minute [bpm]), respiratory rate (RR; breaths per minute), and Modified Behavioral Pain Scale (MBPS) scores. Data are stratified by age-based visit windows: 3–6 months (V1), 12 months (V2), 18 months (V3), and 24 months (V4). The statistical significance of pre- to post-session changes was assessed using Wilcoxon signed-rank tests, with the rank-biserial correlation (r) reported as the measure of effect size. No statistically significant within-group changes were observed.

#### Heart rate

Linear mixed-effects modeling showed no significant pre-to-post change in HR following spTMS (*β* = −0.39 bpm, 95% CI [−4.66, 3.87], *F*(1, 45) = 0.03, *p* = 0.86, η_p_^2^ < 0.01). The overall magnitude of this change was negligible (Cohen’s d = −0.02, 95% CI [−0.25, 0.21]). As a sensitivity analysis, non-parametric Wilcoxon signed-rank tests also found no significant changes within any age group (all *p* > 0.05). Importantly, all HR values remained within age-appropriate normative ranges. Furthermore, post-session HR was not significantly associated with either maximum stimulation strength (*F*(1, 43) = 0.69, *p* = 0.41, η_p_^2^ = 0.02) or the number of pulses delivered (*F*(1, 41) = 0.16, *p* = 0.69, η_p_^2^ < 0.01).

#### Respiratory rate

Linear mixed-effects modeling showed no significant pre-to-post change in RR following spTMS (*β* = −0.61 breaths/min, 95% CI [−3.07, 1.85], F(1, 45) = 0.24, *p* = 0.63, η_p_^2^ < 0.01). The overall magnitude of this change was negligible (Cohen’s d = −0.05, 95% CI [−0.26, 0.16]). As a sensitivity analysis, non-parametric Wilcoxon signed-rank tests also found no significant changes within any age group (all *p* > 0.05). Importantly, all RR values remained within age-appropriate normative ranges. Furthermore, post-session RR was not significantly associated with either maximum stimulation strength (F(1, 43) = 0.40, *p* = 0.53, η_p_^2^ < 0.01) or the number of pulses delivered (F(1, 41) = 3.00, *p* = 0.09, η_p_^2^ = 0.07).

#### Modified behavioral pain scale

Linear mixed-effects modeling showed no significant pre-to-post change in MBPS following spTMS (*β* = 0.39, 95% CI [−0.16, 0.94], F(1, 45) = 1.92, *p* = 0.17, η_p_^2^ = 0.04). The overall magnitude of this change represented a small effect (Cohen’s d = 0.26, 95% CI [−0.12, 0.63]). As a sensitivity analysis, non-parametric Wilcoxon signed-rank tests also found no significant changes within any age group (all *p* > 0.05). Furthermore, post-session MBPS was not significantly associated with either maximum stimulation strength (F(1, 43) = 0.09, *p* = 0.77, η_p_^2^ < 0.01) or the number of pulses delivered (F(1, 41) = 0.00, *p* = 1.00, η_p_^2^ < 0.01).

### Feasibility outcomes

All 46 spTMS sessions were successfully completed with usable bilateral EMG data acquired in all sessions. No sessions were discontinued due to infant distress or procedural challenges. The duration of active TMS assessment, excluding equipment setup and takedown, ranged from 21.56 to 91.32 min, with a median duration of 38.12 min (IQR: 15.25 min).

**Figure 5:**
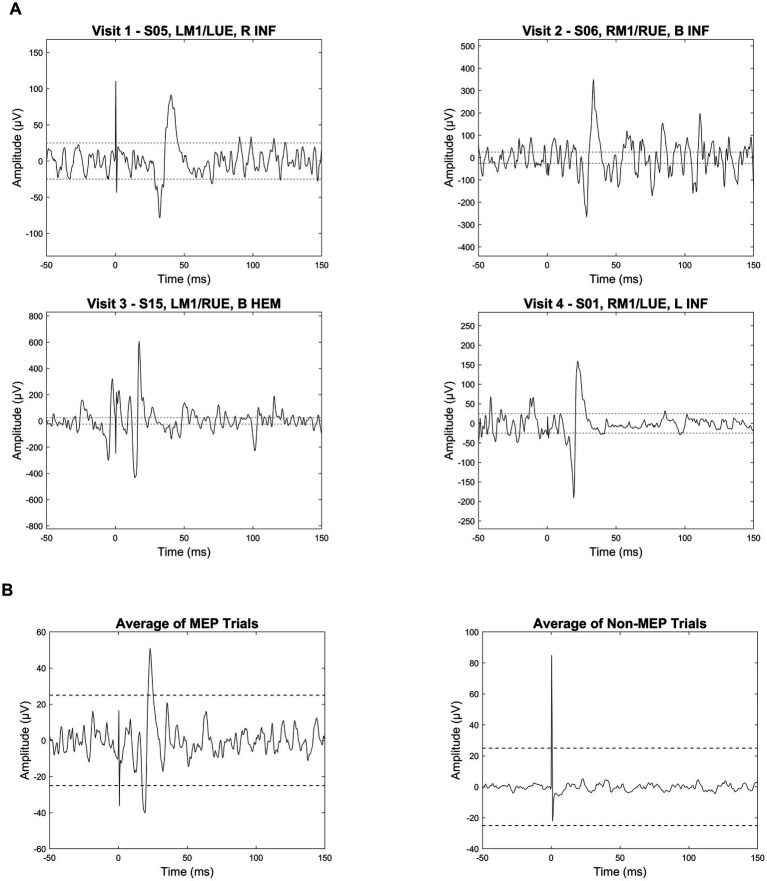
MEP trial examples and average waveforms in infants with perinatal brain injury. **(A)** Individual electromyography (EMG) traces from four infants illustrate examples of motor-evoked potential (MEP) responses following single-pulse transcranial magnetic stimulation (spTMS). Each trace is labeled with the subject ID (e.g., S05), stimulation site (e.g., LM1: left primary motor cortex), target limb (e.g., LUE: left upper extremity), and lesion characteristics. Lesion abbreviations include R (right), B (bilateral), INF (ischemic infarct), and HEM (hemorrhage). Dashed horizontal lines are placed at ±25 μV to visually represent the 50 μV peak-to-peak amplitude threshold used as one criteria to identify a positive motor-evoked potential (MEP). **(B)** Average EMG waveforms are shown for all trials classified as MEPs (left) versus non-MEPs (right). All plots are aligned to stimulus onset (0 ms). Note that vertical scaling differs across plots to optimize waveform visibility.

On average, 54.93 pulses were delivered per session (range: 18–99; IQR: 13.75) at intensities between 50 and 80% MSO. Participant-level data are provided in Table 3. Across all sessions, a total of 5,074 EMG trials were available for analysis, of which 5.9% (301) were scored as positive (POS) based on consensus criteria (see Figure 5 for examples). The rate of positive MEP trials was 8.7% (178/2058) at V1, 4.1% (59/1436) at V2, 3.7% (33/898) at V3, and 4.6% (31/682) at V4. When analyzed by pathway type, contralateral pathways yielded a descriptively higher rate of positive trials (6.4% [162/2537]) than ipsilateral pathways (5.5% [139/2537]), though this difference was not statistically significant (χ^2^(1) = 1.87, *p* = 0.17). Using these trial-level data, we determined that at least one MEP was identified in 95.7% (44/46) of sessions and 95% (19/20) of infants.

**Table 3:** Session-level injury characteristics, stimulation parameters, and motor-evoked potential (MEP) responses.

Participant	Session	Pulses	Max %MSO	Injury type	Injury laterality (L/R/B)	Cerebral palsy			Left hemisphere stim		Right hemisphere stim	
						Dx	Type	Topography	L arm	R Arm	L arm	R arm
S01	V1	55	80	INF	L	N	n/a	n/a	+	+	*—	+
	V2	52	65						+	+	+	+
	V3	40	75						*—	+	+	*—
	V4	76	80						+	+	+	+
S02	V1	47	75	HEM	R	N	n/a	n/a	+	+	+	*—
	V2	74	80						*—	+	+	+
	V3	57	75						+	*—	*—	+
S03	V1	52	80	HEM, HIE	B	N	n/a	n/a	*—	*—	+	+
S04	V1	69	80	PVL	B	Y	S, D, H	n/a	−	−	*—	*—
S05	V1	70	75	INF	R	N	n/a	n/a	+	+	+	+
	V3	42	80						+	*—	*—	+
	V4	58	75						*—	+	*—	+
S06	V1	79	75	INF	B	Y	S	H	*—	+	+	+
	V2	99	80						+	+	+	+
	V3	60	80						+	+	+	+
	V4	62	80						*—	+	+	+
S07	V1	37	65	INF, HEM	B	Y	S	H	+	+	+	+
	V2	46	75						+	+	+	*—
	V3	57	80						+	+	*—	+
	V4	51	80						+	*—	+	+
S08	V1	53	65	HEM	R	N	n/a	n/a	+	+	+	+
	V2	38	80						+	*—	+	+
	V4	39	70						*—	+	*—	+
S09	V1	44	65	HEM	R	N	n/a	n/a	+	+	+	+
S10	V1	51	80	INF, HEM	B	Y	S	H	+	*—	+	+
	V2	54	80						*—	*—	+	*—
	V3	53	75						+	*—	*—	*—
S11	V1	48	70	HEM, PVL	B	N	n/a	n/a	+	+	+	+
	V2	75	80						+	+	+	*—
	V3	42	70						+	+	*—	*—
S12	V1	60	80	HEM, HIE	B	N	n/a	n/a	+	+	+	+
	V2	58	80						+	+	+	*—
S13	V2	57	80	INF, HEM	B	Y	n/a	H	+	*—	+	*—
S14	V1	36	75	PVL	B	Y	S	Q	+	+	+	+
	V2	71	75						*—	+	+	*—
S15	V1	63	80	HEM	B	Y	n/a	H	−	+	*—	+
	V2	54	80						+	+	*—	*—
	V3	81	80						*—	+	−	+
	V4	57	75						*—	*—	*—	*—
S16	V1	39	80	HEM, HIE	B	N	n/a	n/a	*—	*—	+	+
S17	V1	40	75	HEM	B	N	n/a	n/a	+	+	+	+
S18	V1	54	80	INF	L	N	n/a	n/a	−	*—	*—	+
	V2	57	80						*—	+	*—	*—
	V3	18	70						+	+	*—	+
S19	V1	54	80	HIE	B	Y	n/a	n/a	*—	+	+	*—
S20	V1	48	65	HEM	L	N	n/a	n/a	+	+	+	+

Each row represents a single spTMS session (*n* = 46), categorized by visit window: V1 (3–6 months), V2 (12 months), V3 (18 months), and V4 (24 months). Clinical characteristics are defined for each session. Injury laterality is noted as L (left unilateral), R (right unilateral), or B (bilateral). Injury types include ischemic infarct (INF), hemorrhage (HEM), periventricular leukomalacia (PVL), and hypoxic–ischemic encephalopathy (HIE). Cerebral palsy (CP) characteristics include diagnostic confirmation at the time of most recent study visit (Dx: Y/N), motor type (S, spastic; D, dyskinetic; H, hypotonic), and topography (H, hemiplegia; Q, quadriplegia). Stimulation and response parameters include the maximum stimulator output (%MSO), which is the highest intensity used per session. Presence of a motor-evoked potential (MEP) was determined by a consensus review of trial latency, amplitude, and signal clarity. MEPs were then classified as + (Present) if at least one trial was rated consensus-positive (POS); — (Absent-Definite) if no trials were positive and none were ambiguous (NEG-D); or *— (Absent-Uncertain) if no trials were positive but at least one was rated as ambiguous (NEG-U). These MEP classifications are reported for each of the four hemisphere-arm combinations tested: left hemisphere stimulation targeting the left arm (ipsilateral) and right arm (contralateral), and right hemisphere stimulation targeting the left arm (contralateral) and right arm (ipsilateral).

### Factors influencing MEP detection

MEP detection varied significantly with age. Descriptively, the proportion of POS combinations decreased across visits, with rates of 75.0% (57/76) at V1, 64.6% (31/48) at V2, 58.3% (21/36) at V3, and 58.3% (14/24) at V4. A logistic mixed-effects model confirmed this negative association, showing that for each one-month increase in corrected age, the odds of detecting an MEP decreased by 6% (OR = 0.94, 95% CI [0.89, 0.99]; *β* = −0.06; χ^2^(1) = 5.91, *p* = 0.015).

An analysis of stimulation intensity revealed a significant negative association between the maximum MSO reached during a session and the likelihood of detecting an MEP in that same session. For each 1% increase in maximum MSO, the odds of detecting an MEP decreased by 10% (OR = 0.90, 95% CI [0.83, 0.97]; *β* = −0.11; χ^2^(1) = 7.69, *p* = 0.006). Descriptively, for sessions where the maximum MSO was between 60 and 70%, the POS rate was 87.5% (35/40), while for sessions with a maximum MSO between 70 and 80%, the rate was 61.1% (88/144).

### Relationship between brain injury and corticospinal pathways

Overall, POS responses were obtained in 66.8% (123/184) of all hemisphere-hand combinations. When stratified by the CP diagnostic status at the time of the most recent study visit, POS responses were detected in 72.1% (75/104) of combinations from infants without a current CP diagnosis (CP−) and 60.0% (48/80) from those with a CP diagnosis (CP+).

MEPs were detected in hemisphere-hand combinations varied by injury laterality: 64.3% (72/112) for bilateral injuries, 68.8% (22/32) for left-lateralized injuries, and 72.5% (29/40) for right-lateralized injuries. A mixed-effects logistic regression model confirmed that these differences were not statistically significant (χ^2^(2) = 0.89, *p* = 0.64). Similarly, in an exploratory comparison of contralateral pathways, MEP detection rates were not significantly different (Injured: 70.3% [52/74] vs. Uninjured: 61.1% [11/18]; χ^2^(1) = 0.55, *p* = 0.46).

**Figure 6:**
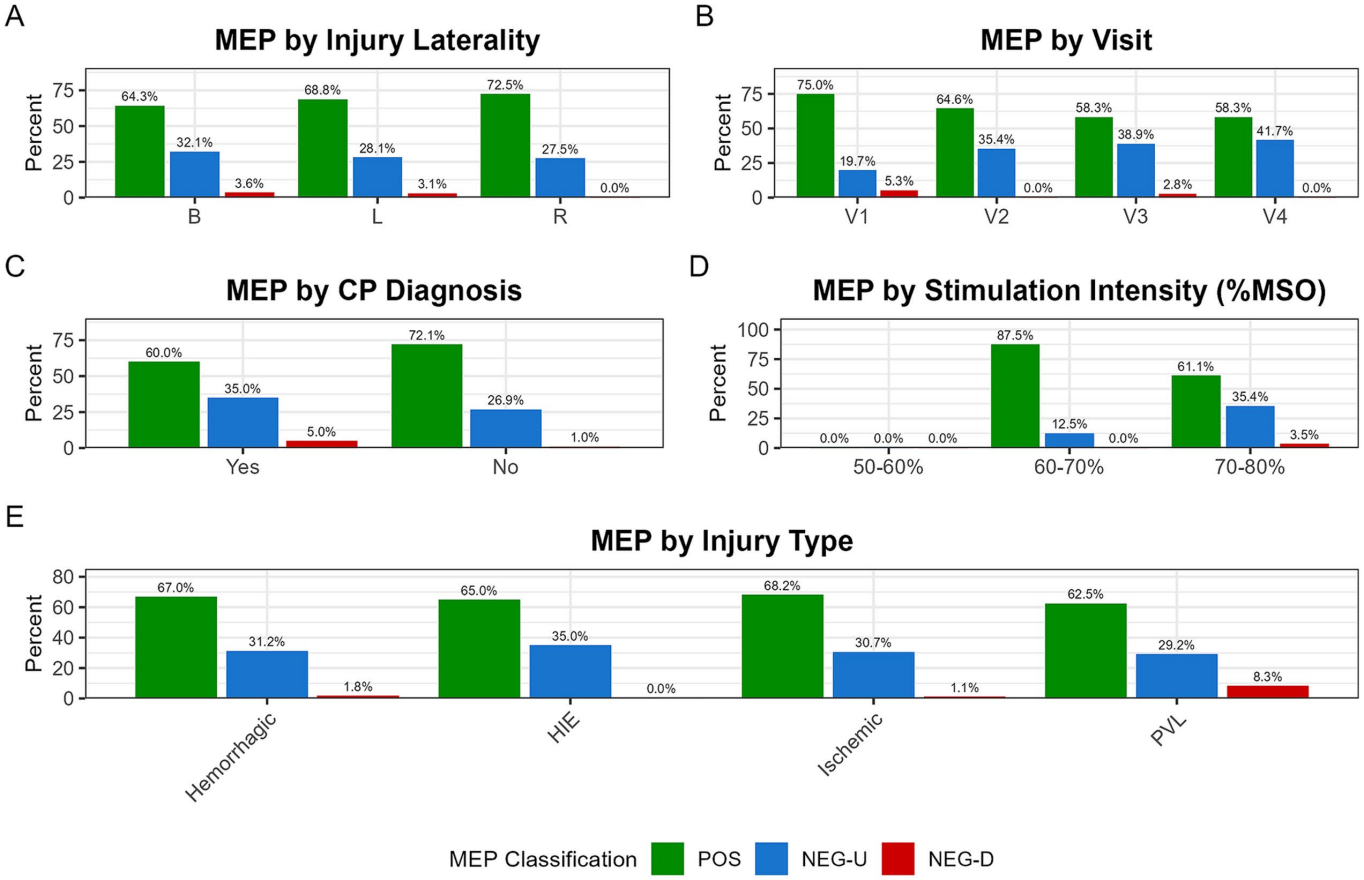
Distribution of MEP classification across clinical and procedural subgroups. Stacked bar plots show the proportion of MEP classifications, which are segmented into MEP-positive (POS; green), MEP-negative with uncertainty (NEG-U; blue), and MEP-negative definite (NEG-D; red). The NEG-U classification was used conservatively when no trials were positive but at least one was ambiguous, preventing misclassification of borderline responses. These distributions are shown across five subgroups: **(A)** injury laterality (B: bilateral, L: left, R: right); **(B)** study visit (V1–V4); **(C)** CP diagnosis at time of most recent study visit (CP+: Yes, CP−: No); **(D)** maximum stimulation intensity (%MSO); and **(E)** injury type (PVL, periventricular leukomalacia; HIE, hypoxic–ischemic encephalopathy).

The absence of a contralateral MEP from an injured hemisphere, a clinically relevant secondary outcome, occurred in 29.7% (22/74) of such hemispheres. The rate of contralateral MEP absence did not differ significantly by injury laterality (Bilateral: 30.4% [17/56], Left: 12.5% [1/8], Right: 40.0% [4/10]; χ^2^(2) = 1.38, *p* = 0.50). However, descriptively, the absence of a contralateral MEP was more common in infants with a current CP diagnosis (35.0% [14/40] of injured hemispheres) compared to those without (23.5% [8/34] of injured hemispheres). Similarly, the absence of a contralateral MEP from an injured hemisphere was common across injury types: HEM (35.4% [17/48]), INF (29.4% [10/34]), PVL (25.0% [3/12]), and HIE (20.0% [2/10]). Descriptive data for these and other clinical subgroups are summarized in Figure 6.

## Discussion

### Key findings—safety and tolerability

Our findings align with previous studies demonstrating that spTMS is safe and well-tolerated in infant cohorts and substantially strengthen this evidence base (Koh and Eyre, 1988; Geerdink et al., 2006; Koudijs et al., 2010; Yang et al., 2013; Gillick et al., 2014; Cassidy et al., 2015; Gillick et al., 2015; Chen et al., 2016; Rich et al., 2016; Rich et al., 2017; Gillick et al., 2018; Keller-Ross et al., 2019; Kowalski et al., 2019; Nemanich et al., 2019; Kowalski et al., 2020; Lixandrão et al., 2020; Rich et al., 2020; Mantell et al., 2021; Narayana et al., 2021; Sutter et al., 2024). Across 46 sessions and 2,527 TMS pulses—a dataset representing a nearly 20-fold increase in the number of pulses delivered within a structured safety framework for this population—no adverse events occurred. We observed no significant physiological changes in heart rate or respiratory rate, with negligible-to-small effect sizes (Cohen’s d = −0.05 to 0.26), underscoring the lack of a clinically meaningful impact. This study provides the first longitudinal analysis of spTMS safety in infants with perinatal brain injury, incorporating a validated behavioral pain scale (MBPS) and repeated auditory screening, which revealed no detectable changes. Longitudinal design is a key contribution, as it enabled ongoing safety monitoring across multiple developmental stages, extending beyond the standard single-session follow-up used in prior work.

### Key findings—feasibility

We were able to successfully enroll 20 infants under the age of two, reflecting the caregivers’ commitment to participate. Importantly, 85% of eligible participants returned for at least one follow-up session, demonstrating sustained engagement and confidence in the study procedures. Attrition occurred primarily for routine longitudinal reasons—loss to follow-up, illness, parental preference, or changes in medical status—rather than spTMS-specific barriers. Furthermore, many families traveled out of town/state distances to attend visits, reinforcing the high degree of motivation to participate in and contribute to this research.

The spTMS procedure demonstrated high feasibility. All 46 planned spTMS sessions were completed in their entirety. This 100% completion rate was matched by a high rate of successful data acquisition. Using pre-defined consensus criteria, MEPs were detected in 95.7% of sessions and 95% of participants. Importantly, these positive responses were distributed across the full spectrum of injury types, laterality patterns, and CP diagnoses, confirming that interpretable neurophysiological data can be acquired under real-world clinical heterogeneity.

Beyond confirming feasibility, our analysis yielded two important insights into factors influencing MEP detection. First, the longitudinal data revealed that the likelihood of detecting an MEP decreased with advancing infant age. This trend may be explained, in part, by the well-established developmental pruning of ipsilateral corticospinal projections. As the motor system matures, these diffuse connections are typically eliminated in favor of predominant contralateral pathways, which would contribute to the negative age-related trend observed in our data (Eyre et al., 2001; Martin, 2005; Eyre et al., 2007; Gordon et al., 2013). However, other factors likely contribute, including the progressive loss of pathways due to the underlying injury, changes in motor threshold due to anatomical or physiological characteristics, and shifts in behavioral state (e.g., voluntary motor control, positioning during spTMS assessment) that change with age. Second, the analysis confirmed a predictable procedural finding: a statistically significant negative association between stimulation intensity and MEP detection. This pattern does not mean higher intensities are less effective. Instead, it reflects the titration protocol, where intensity was only increased when an MEP was not found at lower levels. The statistical result is therefore an expected artifact of the study design, where sessions requiring the highest intensity are, by definition, those in which MEPs were most difficult to elicit.

## Limitations

Our conclusions should be interpreted with several caveats.

### Sample characteristics and generalizability

The primary limitation of this investigation is its relatively small sample size, which may impact the generalizability of the findings. Furthermore, the demographic composition of our sample limits the applicability of the results, as most participants were White and non-Hispanic or Latino. The sample was also geographically and socioeconomically concentrated; most participants resided in metropolitan areas (per RUCA codes), and many were from socioeconomically challenged areas (as indicated by ADI scores). Future studies should prioritize recruiting larger and more diverse samples to validate and extend these findings across all infant populations.

### Challenges in assessing infant tolerability

The tools available to assess infant comfort are imprecise. Safety and tolerability were monitored with the Modified Behavioral Pain Scale (MBPS) together with heart- and respiratory-rate recording. The MBPS, although well established for procedural pain in infants, has not been validated for isolating TMS-specific discomfort; higher scores may just as easily reflect hunger or fatigue as stimulation effects (Crellin et al., 2018b). Likewise, heart- and respiratory-rate stability can miss brief autonomic shifts. Developing neuromodulation-specific infant pain measures, perhaps by adapting instruments such as FLACC or the Non-communicating Children’s Pain Checklist-Revised, remains an important priority (Schiariti et al., 2024). Future work should also incorporate formal assessments of inter-rater reliability for these behavioral scales to further strengthen methodological rigor.

### Interpretation of infant MEP findings

MEP presence or absence must be interpreted cautiously in early infancy. MEPs are shaped by many technical and physiological variables, including stimulation intensity, coil placement, skull penetrance, head size, moment-to-moment fluctuations in cortical or spinal excitability, baseline muscle state or posture, attentional engagement, and the specific muscles sampled (Brasil-Neto et al., 1992; Weissman et al., 1992; Kiers et al., 1993; Darling et al., 2006; Mars et al., 2007; Rösler et al., 2008; Cukic et al., 2009; Keil et al., 2014; Volz et al., 2015; De Goede and Van Putten, 2019; Nemanich et al., 2019; Alawi et al., 2023; Spampinato et al., 2023). For the purpose of documenting feasibility, we labeled a hemisphere-hand combination POS if any single trial was rated as present with moderate-to-high confidence (+1 or +2); in some sessions, this corresponded to one clear response amid predominantly negative or ambiguous trials. Such isolated positives confirm that high-quality EMG data can be captured, but they do not by themselves establish robust CST connectivity. It is also important to distinguish the procedural stopping rule from the analytical feasibility criterion. While our protocol aimed to acquire three MEPs at a given intensity to confirm successful targeting during the session efficiently, our analysis required the presence of a single consensus-scored MEP to classify a session as feasible. This conservative analytical approach was chosen to test the methodological benchmark of successful data acquisition, rather than to infer the functional status of CST connectivity. While this conservative definition was appropriate for demonstrating feasibility, the reliance on a single MEP is not sufficient to infer CST status and underscores the need for consensus standards in infant TMS studies.

Conversely, a negative result at the study’s maximum intensity (80% MSO) does not definitively prove a pathway’s absence. A negative finding may simply reflect a stimulation threshold that exceeds the protocol’s 80% MSO ceiling, particularly since the titration design ensures that only pathways unresponsive at lower intensities are tested at this level. This caution is reinforced by the finding that the vast majority of MEP-negative classifications were designated as NEG-U (91.8% [56/61]), with very few classified as NEG-D (8.2% [5/61]). Until infant-specific guidelines for MEP acquisition, scoring, and interpretation are formalized, these data should be viewed as preliminary indicators rather than conclusive markers of CST organization.

### Exploratory nature of subgroup analyses

The secondary analyses of clinical subgroups should be considered exploratory. This is due both to the novel analytical approach using hemisphere-hand combinations and to the limited statistical power for comparisons involving small groups. For instance, comparisons by injury laterality involved very small groups (e.g., *n* = 3 and *n* = 4 for unilateral injuries), making it difficult to detect true, but modest, effects. Therefore, the non-significant findings for our laterality analyses should be interpreted with caution, as the absence of evidence is not evidence of absence.

### Preliminary nature of CP classification

While we report descriptive statistics stratified by CP diagnosis, these results must be interpreted with caution. A formal CP diagnosis was based on clinical status at the time of the most recent study visit, not on a final longitudinal outcome. The “no CP diagnosis” group is therefore a heterogeneous mix of infants who will not develop CP and those who are simply too young to have been diagnosed yet. Furthermore, the group with a confirmed CP diagnosis may be biased toward infants with more significant injuries or those who were older at the time of assessment, as these factors can lead to an earlier diagnosis. The potential confounds of age and injury severity, rather than diagnostic status alone, may contribute to the observed lower rates of MEP-positive responses in the CP + group. This ambiguity makes direct statistical comparison between the groups inappropriate and limits the conclusions that can be drawn from these descriptive data. Planned long-term follow-up of this cohort will provide definitive motor outcomes, enabling future retrospective analyses that link these early neurophysiological markers to a child’s ultimate functional status.

## Future directions

A significant gap remains in pediatric neurostimulation research. A landmark safety report by Zewdie et al. (2020) underscores this point; the authors highlight that fewer than 4% of over 16,000 neurostimulation studies have included pediatric participants (Zewdie et al., 2020). Their report also reveals the specific need for more research in the youngest populations; while providing critical safety data across a large pediatric cohort, their work included one study with infants and toddlers (*n* = 12; age range: 0.8–4 years, median: 2 years), underscoring the opportunity for future investigations in this crucial developmental window.

Establishing the safety and feasibility of spTMS in infancy provides the foundational starting point for its use as a translational pediatric tool. The present work offers a safe and replicable framework for applying spTMS repeatedly to infants at high risk for CP, opening the door for longitudinal, multimodal investigations needed to guide clinical care. Achieving the statistical power to identify biomarkers and inform interventions through such ambitious work will require multi-site collaborations. These future studies can now build upon the secure methodological platform established here to advance evidence-based guidelines for infant spTMS.

A critical next step for the field is to move beyond demonstrating simple feasibility toward standardizing methods for scientific inference. A key strength of our approach is the use of a rigorous, multi-rater consensus process designed to reliably identify MEPs from noisy infant EMG data. A formal analysis that quantifies the good reliability of this method is presented in a separate manuscript (Casey et al., 2025). Our feasibility criterion—requiring only a single confident MEP—was an intentionally conservative benchmark chosen to establish the success of our data capture methods. This approach was necessary because acquiring a clean neurophysiological signal in our high-risk clinical cohort is a primary methodological challenge in itself.

This focus contrasts with the scientific aims of pioneering developmental studies, such as those by Eyre and colleagues, which were designed to compare developmental trajectories between healthy infants and those with perinatal injury (Eyre et al., 2001; Eyre et al., 2007). Their goals of interrogating CST development necessitated more demanding criteria, such as defining motor threshold as the intensity needed to evoke MEP in 50% of trials and collecting 20 to 30 responses to characterize a pathway. Such criteria are essential for scientific interrogation but are premature for a foundational study focused first on establishing a safe and tolerable framework for data acquisition in a clinically heterogeneous population. Therefore, the priority is to bridge this gap: using the reliable detection framework established here to determine what constitutes a robust and scientifically meaningful measure of CST connectivity in infants with perinatal brain injury. This standardization effort must also include infant-specific protocol optimization—such as secure yet flexible positioning, motion-mitigation, and precise targeting—and the validation of behavioral scales capable of isolating TMS-specific discomfort.

With standardized methods in place, future research can focus on critical questions about motor pathway development. For instance, the high rate of absent contralateral MEPs from injured hemispheres could be validated as an early biomarker for identifying infants at heightened risk for poor motor outcomes. Further work should also probe the anatomical and maturational determinants of MEP detectability, including lesion characteristics, skull thickness, and myelination status. Our finding of an overall decrease in MEP detection with age points to another key question: future studies with larger samples are needed to formally test if this effect is driven primarily by the developmental pruning of ipsilateral projections, a hallmark of motor system maturation.

## Conclusion

This study provides a comprehensive demonstration of the safety, tolerability, and feasibility of single-pulse TMS in infants with perinatal brain injury. In 46 sessions involving 2,527 pulses we observed no adverse events, no meaningful physiological deviations, and only minimal behavioral distress, while achieving complete session completion and strong longitudinal retention. Interpretable EMG signals were captured bilaterally in every session, and at least one moderate-to-high-confidence MEP was recorded in 95.7% of sessions and 95% of participants, demonstrating clear feasibility for neurophysiological data collection at this age.

Because our feasibility criterion required only a single confident MEP, the present findings should not be taken as direct evidence of robust corticospinal connectivity. Instead, they mark an essential methodological milestone: reliable, well-tolerated acquisition of infant EMG responses under rigorously monitored conditions. These findings establish a reproducible framework for the safe and scalable use of spTMS in infancy. Future progress depends on two parallel efforts: (i) refining infant-specific comfort measures and stimulation techniques, and (ii) developing consensus guidelines for how many MEPs, at what confidence and consistency, are needed to infer CST status during early development. With those advances in place, longitudinal spTMS—integrated with imaging and behavioral assessments—has strong potential to yield early biomarkers of motor-pathway health and to guide the timing and targeting of neurorehabilitative interventions in this uniquely plastic developmental window.

## Data Availability

The original contributions presented in the study are included in the article, further inquiries can be directed to the corresponding author.
